# Alterations and correlations in dental plaque microbial communities and metabolome characteristics in patients with caries, periodontitis, and comorbid diseases

**DOI:** 10.1186/s12903-023-03785-3

**Published:** 2024-01-25

**Authors:** Ying Wang, Fei Yang, Yuan Wang, Shuli Deng, Rui Zhu

**Affiliations:** 1 Stomatology Hospital, School of Stomatology, Zhejiang University School of Medicine, Zhejiang Provincial Clinical Research Center for Oral Diseases, Key Laboratory of Oral Biomedical Research of Zhejiang Province, Cancer Center of Zhejiang University, Engineering Research Center of Oral Biomaterials and Devices of Zhejiang Province, Hangzhou, 310000 China; 2 Department of Stomatology, Affiliated XiaoShan Hospital, Hangzhou Normal University, Hangzhou, 310000 China; 3 Department of Nutrition and Food Hygiene, School of Public Health, School of Medicine, Zhejiang University, 866 Yu-hang-tang Road, Hangzhou, Zhejiang Province 310058 China

**Keywords:** Dental Plaque, Microbial, Caries, Periodontitis, Comorbid Diseases

## Abstract

**Backgrounds:**

The pathogenic microorganisms and clinical manifestations of caries and periodontitis are different, caries and periodontitis are usually discussed separately, and the relationship between them is ignored. Clinically, patients prone to dental caries generally have a healthier periodontal status, whereas patients with periodontitis generally have a lower incidence of dental caries. The relationship between dental caries and periodontitis remains unclear.

**Objectives:**

This study aimed to explain the clinical phenomenon of antagonism between dental caries and periodontitis by exploring the ecological chain and bacterial interactions in dental caries, periodontitis, and other comorbid diseases.

**Methods:**

The dental plaque microbiomes of 30 patients with oral diseases (10 each with caries, periodontitis, and comorbid diseases) were sequenced and analysed using 16 S rRNA gene sequencing. The Kyoto Encyclopaedia of Genes and Genomes (KEGG) database was used for a differential functional analysis of dental plaque microbial communities in caries, periodontitis, and comorbid diseases.

**Results:**

The coinfection group had the greatest bacterial richness in dental plaque. The principal coordinate analysis showed that caries and periodontitis were separate from each other, and comorbid diseases were located at the overlap of caries and periodontitis, with most of them being periodontitis. Simultaneously, we compared the microbiomes with significant differences among the three groups and the correlations between the microbiome samples. In addition, KEGG pathway analysis revealed significant differences in functional changes among the three groups.

**Conclusions:**

This study revealed the composition of the dental plaque microbial communities in caries, periodontitis, and comorbidities and the differences among the three. Additionally, we identified a possible antagonism between periodontitis and caries. We identified a new treatment strategy for the prediction and diagnosis of caries and periodontitis.

**Supplementary Information:**

The online version contains supplementary material available at 10.1186/s12903-023-03785-3.

## Introduction

The human body contains diverse microbiomes, which include bacteria, viruses, and fungi [1]. Dental caries and periodontitis, the two most common microbial diseases [2], pose a significant burden on humans. Dental caries is induced by acid production via dental biofilms (dental plaque), which are exposed to sugars, leading to localised chemical dissolution of the tooth surface [3]. Periodontitis is an inflammatory and immunoreactive disease caused by plaque biofilm [4]. Dental biofilms play a major biological role in the progression of both diseases [3]. Dental biofilms are plaques composed of colonies of various bacteria, algae, fungi, and debris [5].

The clinical manifestations are widely different in caries and periodontitis; therefore, researchers usually discuss caries and periodontitis separately and may ignore the relationship between these two diseases. Views differ regaring the relationship of caries and periodontitis with dental plaque biofilms. some researches revealed suggested that there is a correlation between occurrence of both dental caries and periodontitis [6]. The patients suffered from higher attachment loss and probing depths at sites with caries experience compared to sites without caries experience [3]. On the other hand, Plaque components and clinical manifestations differ between caries and periodontitis [7]. Bacteria associated with dental caries and periodontitis produce antagonistic effects [8]. Caries and periodontitis are independent [9].

A potential correlation exists between dental caries and periodontitis [6]. Patients with caries exhibit higher attachment loss and probing depth at sites affected by the condition than that exhibited by those without caries [3]. We do not yet know.

The microbiome in periodontitis and dental caries is dramatically altered, as shown by sequencing, indicating the possibility of a microbial existence. The analysis of functional differences can provide information on microbial functions [3].

The aim of the present study was to compare dental biofilms in patients with caries, periodontitis, and comorbid diseases using 16 S V4 DNA sequencing. We explored the relationship between the oral microbiome in caries and periodontitis, which could facilitate screening of these two oral diseases related to dental biofilms.

## Materials and methods

### Study population and sample collection

We recruited 30 individuals (age: 18–60 years) from the Stomatology Hospital, Zhejiang University School of Medicine, from March 2022 to April 2022. The inclusion criteria were: [1] no history of periodontal treatment in the past 6 months or antibiotic intake within the past 3 months and [2] absence of systemic disease, pregnancy, or lactation. To exclude the influence of obesity on periodontitis, we ensured that the participants weighed within the normal range (18.5 kg/m²≤ body mass index < 24 kg/m²). All participants agreed to participate in the study, signed an informed consent form, and were categorised into the following three groups: periodontitis (n = 10), dental caries (n = 10), and comorbid diseases (caries and periodontitis; n = 10). A single physician performed all oral clinical examinations. Dental biofilms (dental plaque) were collected from concentrated particles using disposable swabs(Medico, Shenzhen, China). The swabs were brushed at the collection sites 3 or 4 times, and the other side of the swabs was used to repeat the aforementioned operations. Saving the head of swabs containing dental biofilms in an 1.5 ml sterile EP tube (Axygen,CA,USA) encapsulating 200 mL of sterile normal saline (Biosharp, Hefei, China). Swabs were transported to the local laboratory within 2 h, and stored at -80 °C until DNA extraction.

### DNA isolation, polymerase chain reaction (PCR) amplification, and 16 S rRNA gene sequencing

Microbial community DNA was extracted from the tool samples using the MagPure Stool DNA KF Kit B (Magen, China) following the manufacturer’s instructions. Furthermore, for the PCR, 30 ng of the qualified DNA template and variable regions V3–V4 of bacterial 16S rRNA primers (341F (5’-ACTCCTACGGGAGGCAGCAG-3’) and 806 R (5’- GACTACHVGGGTWTCTAAT-3’) were added. PCR cycling conditions were as follows: 94 °C for 3 min, 30 cycles of 94 °C for 30 s, 56 °C for 45 s, 72 °C for 45 s, and a final extension for 10 min at 72 °C for 10 min. The PCR products were purified using AmpureXP beads and eluted in ion buffer. Libraries were constructed using an Agilent 2100 Bioanalyzer (Santa Clara, CA, USA). The validated libraries were sequenced on an Illumina MiSeq platform (BGI, Shenzhen, China) following the standard Illumina pipeline, generating 2 × 300 bp paired-end reads.

### Sequencing data processing and statistical analysis

Raw reads were filtered and paired-end reads were added to tags using the Fast Length Adjustment of Short reads programme (FLASH, v1.2.11). The tags were clustered into Operational taxonomic units(OTUs)with a cutoff value of 97% using the UPARSE software (v7 0.0.1090). OTU sequences were taxonomically classified using the Ribosomal Database Project Classifier v.2.2, with a minimum confidence threshold of 0.6.

The alpha and beta diversities were estimated at the OTU level using MOTHUR (v. 1.31.2) and QIIME (v. 1.8.0) at the OTU level. Based on UPGMA, a sample cluster was constructed using QIIME (v. 1.8.0). Kyoto Encyclopaedia of Genes and Genomes (KEGG) functions could further explain the role of the targets in metabolism, signal transduction and other processes [10]. KEGG functions were analysed using PICRUSt software. Barplot and heatmap of different classification levels were plotted with R package v. 3.4.1 and R package ‘gplots’, respectively. The Venn plots in OTUs or taxa were plotted with R package.

‘VennDiagram’ version 3.1.1. The OTU rank curve was plotted using R package version 3.1.1. Principal coordinate analysis (PCoA) was performed using QIIME (v. 1.8.0). Significant species or functions were determined by R (v. 3.4.1) based on Wilcox or Kruskal test.

## Results

### Microbial composition among the groups

Thirty samples were sequenced and 1,755,392 high-quality sequences were obtained, with an average of 58,513 sequences per sample. We clustered the 16 S rRNA gene sequences into OTUs and detected 24 phylum, 48 classes, 85 orders, 139 families, and 215 genera.

**Fig. 1:**
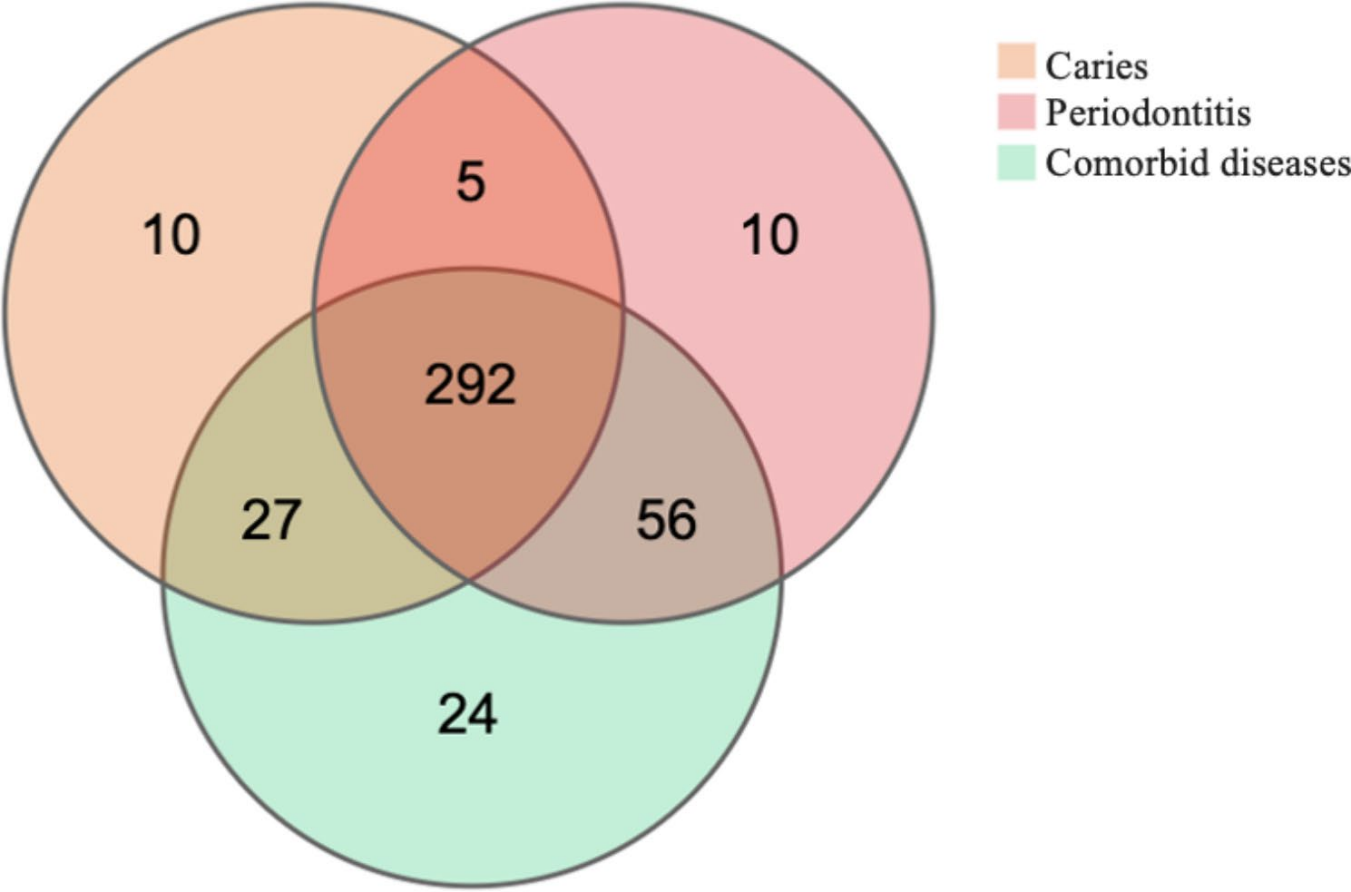
Venn diagram of caries, periodontitis, and comorbid diseases. Different colours in the figure represent different samples or groups, and the numbers in the pairwise overlap part are the number of OTUs common between two samples or groups. Similarly, the number in the overlapping part indicates the number of OTUs common among multiple samples or groups

For the rarefaction curve, Good’s coverage estimator for each sample approached almost 100% (> 99.7%), indicating that the sequencing depth was sufficient to reflect most bacterial characteristics of the samples (Appendix 1). To determine the microbiota composition in each group, Venn plots were used to identify unique and common OTUs. The results revealed 334, 363, and 379 OTUs for caries, periodontitis, and comorbid diseases, respectively. Caries and periodontitis contained 10 unique OTUs, whereas comorbid diseases contained 24 OTUs (Fig. 1).

### Bacterial diversity of caries, periodontitis, and comorbid Diseases

**Fig. 2:**
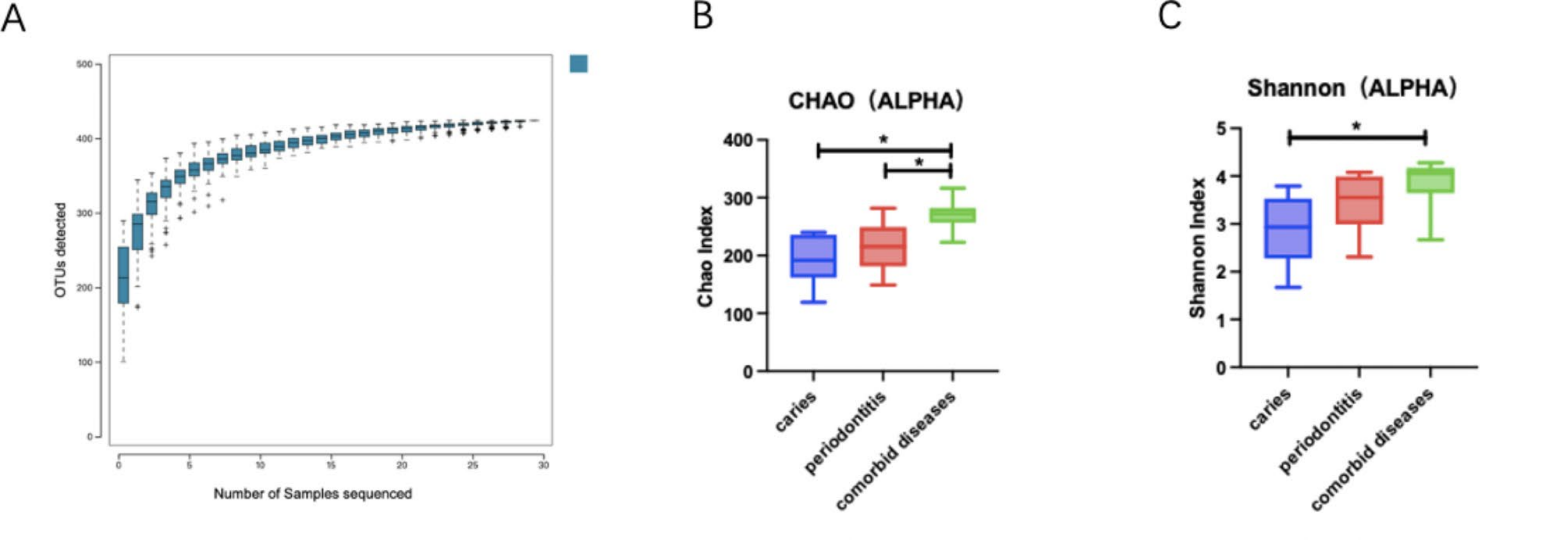
Alpha diversity analysis of caries, periodontitis, and caries-active and comorbid diseases. (**A**) Species accumulation curve (**B**) Chao1 index and (**C**) Shannon index of each group. *p < 0.05

The species accumulation curves flattened at the ends, indicating that the sampling numbers were adequate (Fig. 2A). Shannon and Chao1 indices were used to measure alpha diversity. Comorbid diseases were associated a higher Chao1 index than those associated with caries and periodontitis, indicating that samples from the coinfection group had higher bacterial richness (Fig. 2B). The Shannon index of fine bacterial density and evenness did not differ significantly between comorbid diseases and periodontitis. However, comorbid diseases and caries differed significantly (Fig. 2C).

### Changes in microbial composition

**Fig. 3:**
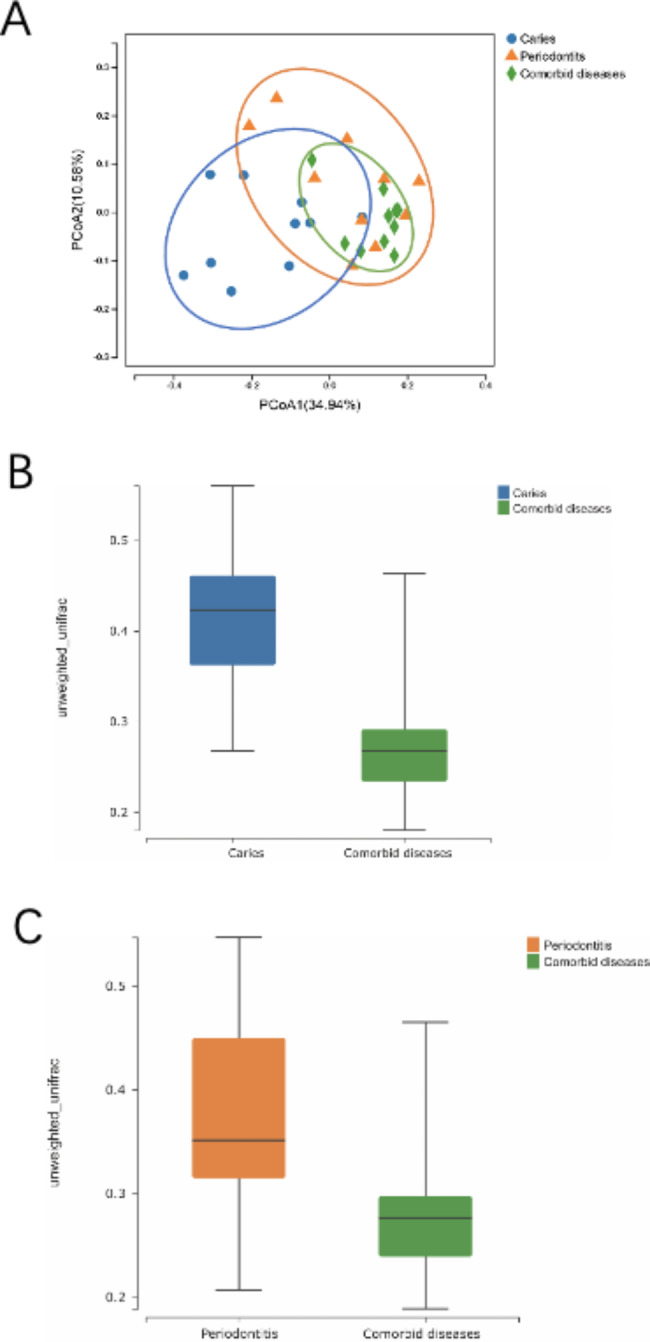
Principal coordinate analysis (PCoA) and beta diversity differences among caries, periodontitis, and comorbid diseases: (**A**) PCoA plots showing the separation of samples from caries, periodontitis, and comorbid diseases. (**B** and **C**) Plots of group differences in beta diversity of caries and comorbid diseases and periodontitis and comorbid diseases. *p < 0.05

PCoA and plots of group differences in beta diversity based on unweighted UniFrac distances were performed to determine changes in microbial composition between the groups. A proper separation was between caries and periodontitis, indicating that the microbial community composition of caries and periodontitis differed. Comorbid diseases partially overlapped with caries; however, most were associated with periodontitis (Fig. 3A). The beta diversity differed between caries and comorbid diseases and between periodontitis and comorbid diseases (Fig. 3B and C).

### Microbiological compositions of caries, periodontitis, and comorbid Diseases

**Fig. 4:**
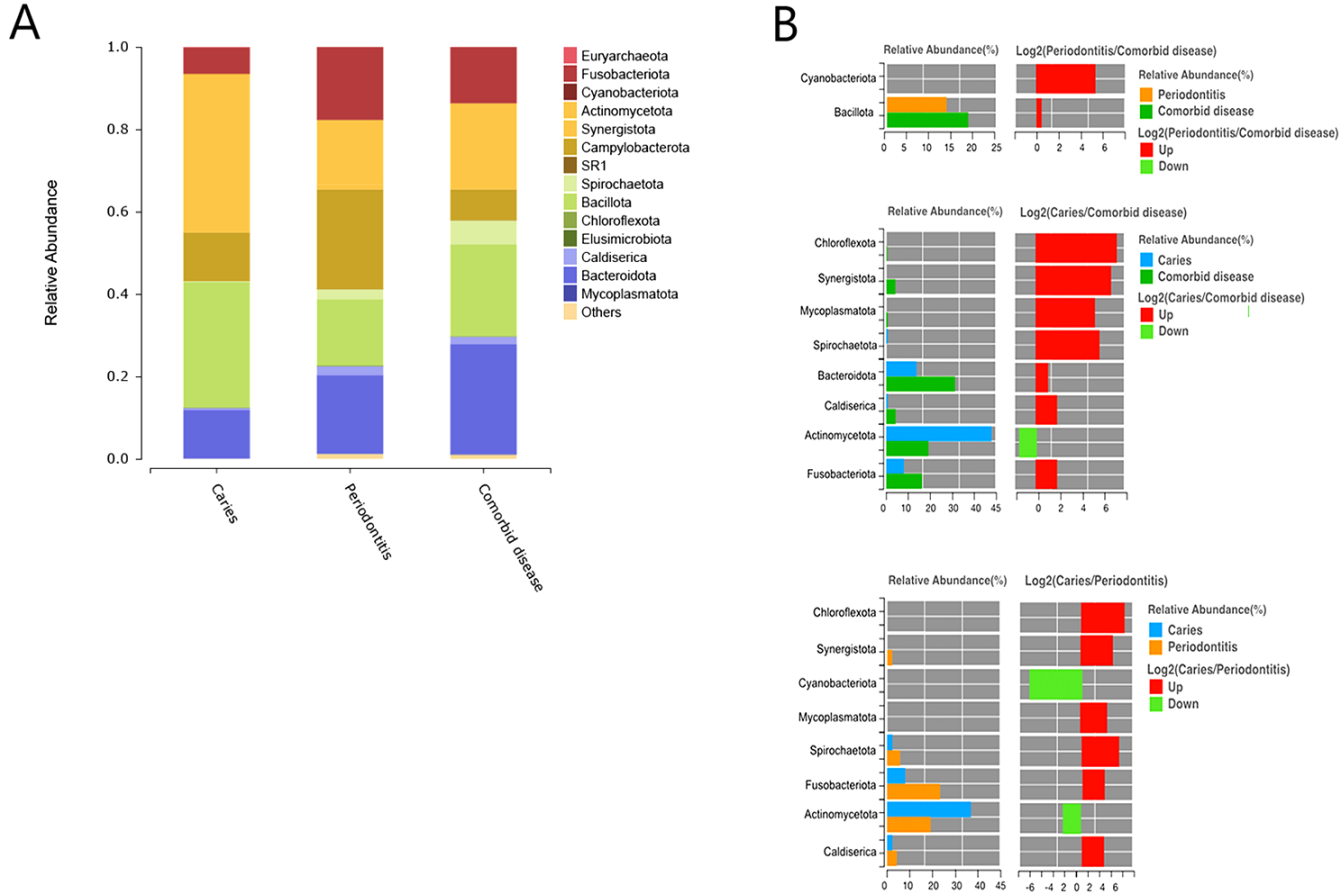
Analysis of microbial profiles among caries, periodontitis, and comorbid diseases. (**A**) Relative abundance levels of the most dominant phylum among caries, periodontitis, and comorbid diseases. (**B**) Analysis of species differences among caries, periodontitis, and comorbid diseases

The species composition analysis at the phylum level showed that Actinomycetota, Bacillota, Fusobacteriota, Pseudomonadota, and Bacteroidota were the five most common dominant phylum, accounting for > 95% of the total sequences. Actinomycetota accounted for nearly 40% of caries cases, 15.8% of periodontitis cases, and 18.0% of comorbid diseases. The changing trend of Bacillota in the three groups was consistent with that of Actinomycetota, which was 30.1%, 16.1%, and 22.4%, respectively. Fusobacteriota accounted for 6.4% of caries, 17.7% of periodontitis, and 13.6% of comorbid diseases Pseudomonadota accounted for the highest proportion in periodontitis (24.2%), caries (11.8%), and comorbid diseases (7%). Bacteroidota accounted for 11.8%, 19.1%, and 26.6% of the caries, periodontitis, and comorbid diseases, respectively. Spirochaetota, Synergistota, and Candiserica accounted for nearly 0% of caries but more than 1% of periodontitis and comorbid diseases (Fig. 4A). The analysis of species differences between caries and periodontitis revealed the presence of Chloroflexota, Synergistota, and Cyanobacteria.Mycoplasmatota, Spirochates, Fusobacteriota, Actinomycetota, and Candiserica differed significantly (*p* < 0.05). The analysis of species differences between caries and comorbid diseases showed that Chloroflexota, Synergistota, Enericutes, Spirochates, Fusobacteriota, Actinomycetota, and *Candiserica* were significantly different (*p* < 0.05). The analysis of species differences between periodontitis and comorbid diseases showed that Cyanobacteriota, Bacillota, and Bacteroidota were significantly different (*p* < 0.05; Fig. 4B).

### Correlation analysis of caries, periodontitis and comorbid Diseases

**Fig. 5:**
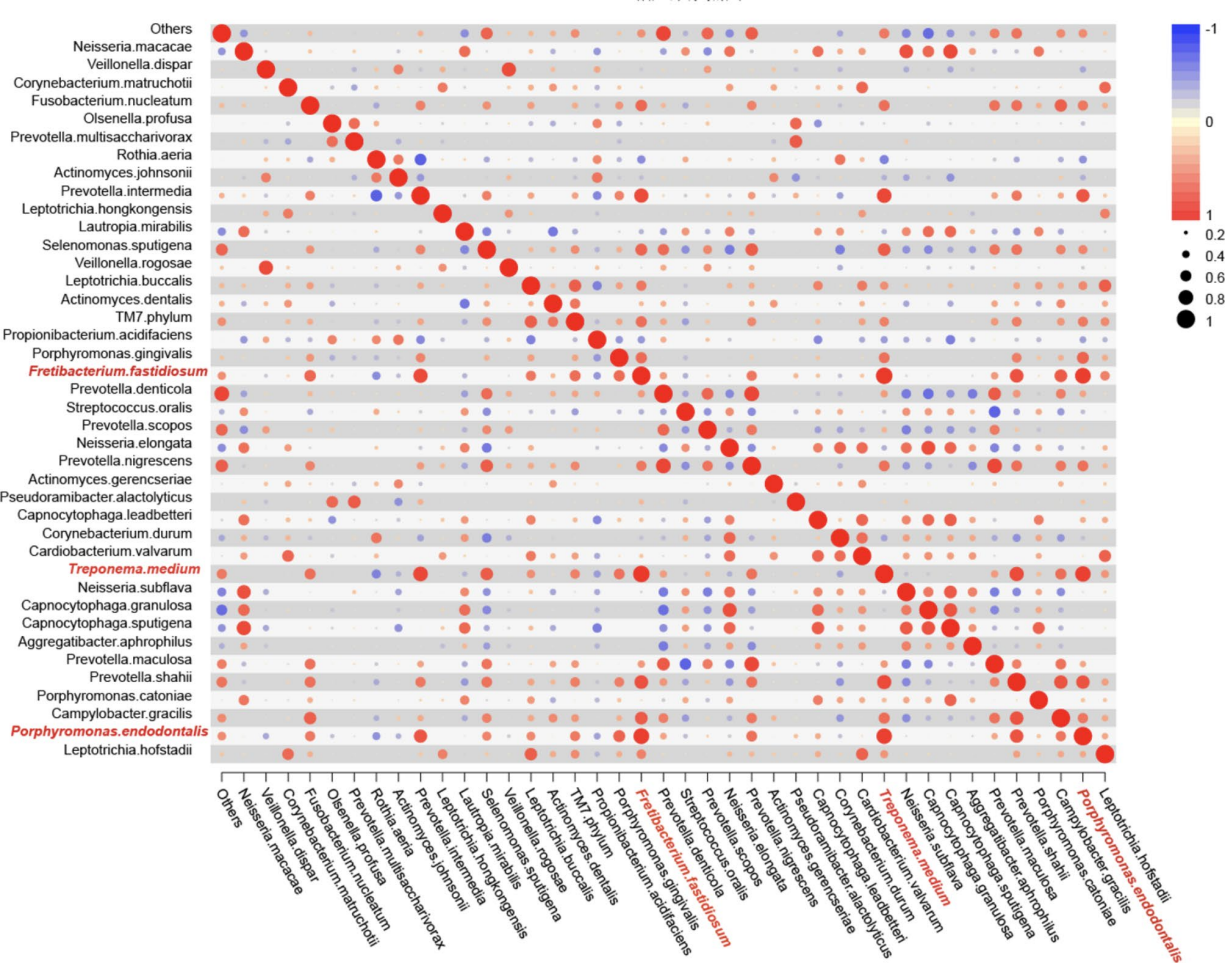
Correlation analysis among caries, periodontitis, and comorbid diseases. The colour of the circles represents the direction of correlation, and their size corresponds to the significant value of *p* (The larger the circle, the smaller the p-value, and the higher the significance)

The ecological associations and interactions among different genera were further investigated using Pearson’s correlation analysis (Fig. 5). Treatment with *Treponema medium, Freitobacterium fastidiosum*, *Porphyromonas endodontalis* were positively correlated with each other. The coefficient of the positive correlation was greater than 0.85.

### Functional differences in microbiota of caries, periodontitis and comorbid Diseases

**Fig. 6:**
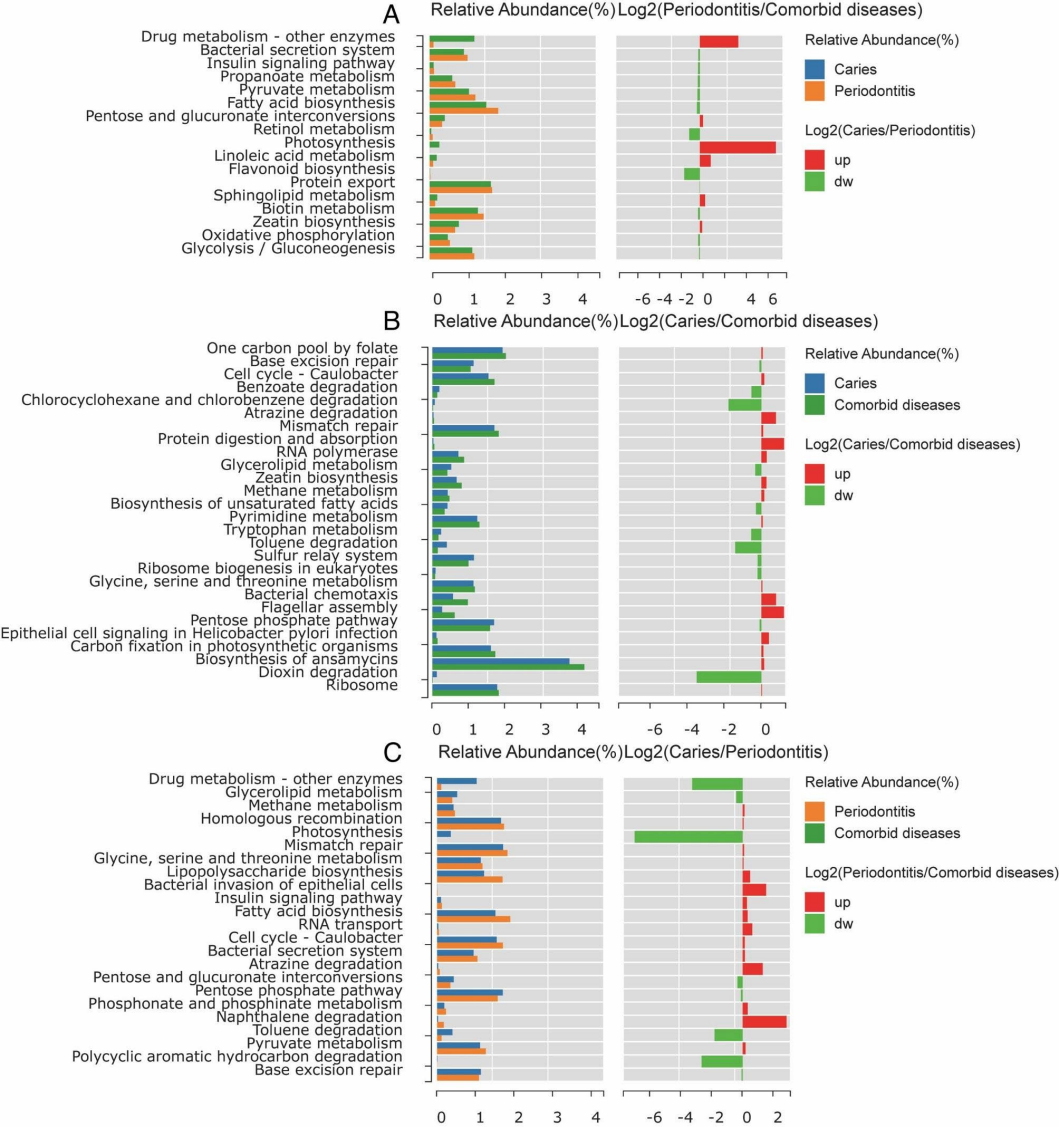
Functional changes in microorganisms among caries, periodontitis, and comorbid diseases. Prediction and analysis of microbial functional pathways based on the KEGG database. Significantly differential functions between (**A**) periodontitis and comorbid diseases, (**B**) caries and comorbid diseases, and (**C**) caries and periodontitis are shown (*p* < 0.05)

Differences in microbial function can lead to the occurrence and development of different diseases. Therefore, we used the KEGG pathway analysis to describe the differences in biological functions among the three groups [11]. Compared to periodontitis, we identified significant differences in 23 pathways in caries, and the relative abundances of seven pathways decreased (Fig. 6A). Comorbid diseases exhibited 19 pathways with significant differences, as opposed to periodontitis, wherein seven pathways were associated with increased relative abundance (Fig. 6B). Compared to comorbid diseases, 27 pathways differed significantly in caries, among which 11 were associated with a reduced relative abundance (Fig. 6C).

## Discussion

Dental caries and periodontitis, which are the most common oral diseases, have been the focus of oral research. The oral microbial composition differs between the two diseases. However, the association between dental caries and periodontitis requires further investigation.

Dental biofilms play a major biological role in the progression of both diseases [3]. Once plaque forms, the species composition at a site is characterised by the degree of stability or balance between the constituent species [12]. Subtle intercellular signalling may also occur, leading to coordinated gene expression within microbial communities [13, 14]. Furthermore, close contact between microorganisms and dental plaques increases the possibility of microbial interactions, thus regulating the caries and periodontitis potential of pathogenic bacteria [4, 15]. Compared to the attachment of saliva, dental plaque exhibits microbiomes attached to the hard tissue surface more intensively, while avoiding the interference of the microbiome on the soft tissue surface [16]. Supragingival and subgingival plaques correlated with oral microorganisms rather than saliva in identifying caries. Periodontitis-related microorganisms are the most common [17]. To eliminate differences in microbial and metabolic compositions and pathways in different ecological niches, the mesial gingival margin on the palatal side of the maxillary first molar was used as a unified sampling point, and the supragingival plaque was scraped from this site.

We chose V3-V4 as the region for this study. Due to the longer amplicon size of the V1-V3 region compared to V3-V4, the merge rate of the paired-end reads is higher in the V3-V4region than in the V1-V3region [18]. And many recent studies reported that V3-V4 regions could be used for oral microbiota studies [19–21].

The correlation analysis revealed the presence of *Treponema medium, Freitobacterium fastidiosum*, *Porphyromonas endodontalis*. These bacteria exhibited a strong correlation and were the main pathogenic bacteria in the periodontitis group [22–24]. Plaque at the sampling site reflected the composition of subgingival plaque to a certain extent. We observed no significant differences in microbial abundance or diversity between the periodontitis and the combined groups. In contrast, the caries group had lower microbial abundance and diversity compared to those exhibited by the periodontitis and combined groups. This might be related to the antagonism effects between dental caries and periodontitis produce antagonistic effects [8]. However, a previous report focused on oral saliva as the research object [7], which further confirmed that the energy substances metabolised by dental plaque flora mainly come from saliva and that saliva accepts microorganisms flowing from dental plaque. In conclusion, dental plaque at the gingival margin reflects the microbial composition of the upper and lower gingival plaques to a certain extent. PCoA and beta diversity analysis showed that caries and periodontitis were separated from each other, and comorbid diseases were located in the overlapping area between the first two groups. Shi C et al. reported showed that caries and periodontitis groups were separated from each other, suggesting the structural difference of their salivary microbial communities [7], consistent with the present results. It may partially explain the phenomenon that after periodontal therapy, prevalence of dental caries increased [9]. The results reminded us that a stricter plaque control at the gingival margin should be performed during periodontal caries therapy and a stricter caries preventive program should be performed during initial periodontal therapy.

Actinomycetota, Bacillota, Fusobacteriota and Pseudomonadota did not increase with the concurrent development of the two diseases. Therefore, we hypothesised that changes in the microbiome may cause the antagonistic manifestations of these two diseases. Changing the composition of the microbial community in the dental plaque using probiotics is beneficial for oral health. These results provide a new theoretical basis for treating diseases using probiotics [25].

Functional differences between periodontitis and comorbid diseases were analysed to clarify the reasons for low caries rates in patients with periodontitis. Sphingolipid metabolism was altered in patients with caries-complemented periodontitis. Sphingolipids can destroy biofilms containing *Streptococcus mutans* [26], the main microbes that cause dental caries [27]. Photosynthesis is an important metabolic pathway in *Cyanobacteria*, and its metabolites include potent toxins [28]. Similarly, among the significantly different genes in caries and periodontitis, the relative abundance of Cyanobacteriota was higher in caries than in periodontitis. However, the relative abundances of Cyanobacteriota in caries and comorbid diseases were not significantly different. The results of this study were similar to those of previous studies, and the relative abundance of Cyanobacteriota was the highest in the group with caries. Most Cyanobacteriota are highly toxic to sulphides [29], which can aggravate periodontitis [30]. Therefore, we speculate that the low incidence of dental caries in patients with periodontitis may be related to the oral microbial environment of patients with periodontitis, which inhibits the growth of Cyanobacteriota.

Additionally, we observed a downregulation in the relative abundance of periodontitis accompanied by dental caries and flavonoid metabolism. Recent research indicates that oral biofilms prevent dental caries [31] by inhibiting *Streptococcus* community growth in dental plaque biofilms [32]. Pyruvate metabolism regulates hydrogen peroxide production and acid tolerance in various oral *Streptococcus* species, thereby affecting the occurrence of dental caries [33]. Therefore, changes in pyruvate metabolism may contribute to the lower incidence of caries in patients with periodontitis.

To identify the possible reasons for the low incidence of periodontitis in patients with caries, a functional analysis was performed to examine the relationship between caries and comorbid diseases. When periodontitis is complicated by caries, the relative abundance of RNA transport, riboflavin metabolism, and ribosome biogenesis in eukaryotes increases, indicating that bacteria grow more rapidly, which might be influenced by periodontitis. During periodontitis, the gingival sulcus exudates, providing nutrients for bacterial growth and reproduction [34]. Similarly, the relative abundances of flagellar and bacterial assemblies increased, with the flagellar assembly being essential for the movement of Spirochaetota in sticky environments and for increasing the chemotaxis of the bacteria. Spirochaetota are the main pathogens responsible for periodontal diseases [35, 36]. Therefore, metabolism *of* Spirochaetota remains the main cause of periodontal diseases. Significant differences were observed between Spirochaetota and other genera. The relative abundance of benzoate degradation is higher in caries than in comorbid diseases, and the increased secretion of benzoate is conducive to the reproductive division of Spirochaetota [37]. Therefore, the oral microbiome of patients with caries may prevent periodontitis by inhibiting benzoic acid secretion.

## Conclusion

Significant differences were observed in the microbial communities of dental plaques among different groups. Each group has its specific high-abundance bacteria, which may have important clinical implications for the screening and early treatment of individuals with dental caries and periodontitis. The metabolites of oral microorganisms in patients with periodontitis may inhibit the reproduction of caries-causing microorganisms. Similarly, the metabolites of oral microorganisms in patients with caries may inhibit the reproduction of pathogenic microorganisms in periodontitis. In addition, caries - associated bacteria and periodontitis - associated bacteria can co-exist. These findings explain the antagonistic relationship between periodontitis and caries and provide a new treatment strategy for predicting and diagnosing these diseases. Further studies are warranted to evaluate if screening of microbial activity of specific oral bacterial species can identify periodontitis and dental caries at preclinical stages.

### Electronic supplementary material

Below is the link to the electronic supplementary material.

Supplementary Material 1

Supplementary Material 2

## Data Availability

The data are available upon request. All data sharing and collaboration requests were directed at the corresponding author (Rui Zhu, 625,515,637@qq.com).
