# Supplementary figures and images for: Alterations and correlations in dental plaque microbial communities and metabolome characteristics in patients with caries, periodontitis, and comorbid diseases
Source: BMC Oral Health. 2024 Jan 25;24:132. doi: 10.1186/s12903-023-03785-3 (PMC10811826; doi:10.1186/s12903-023-03785-3)

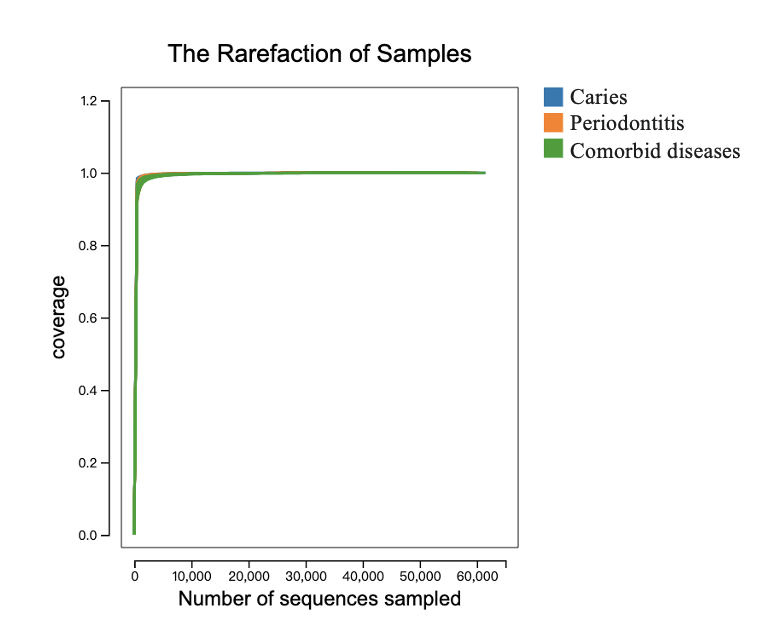

Supplement: Supplementary file 1 — Supplementary Material 1 [file 12903_2023_3785_MOESM1_ESM.png]
