# Supplementary material for: Alterations and correlations in dental plaque microbial communities and metabolome characteristics in patients with caries, periodontitis, and comorbid diseases
Source: BMC Oral Health. 2024 Jan 25;24:132. doi: 10.1186/s12903-023-03785-3 (PMC10811826; doi:10.1186/s12903-023-03785-3)
Supplement: Supplementary file 2 — Supplementary Material 2 [file 12903_2023_3785_MOESM2_ESM.docx]

| sample_name | tag_num | otu_num |
| --- | --- | --- |
| Caries 1 | 57205 | 212 |
| Caries 2 | 58275 | 185 |
| Caries 3 | 59376 | 129 |
| Caries 4 | 57537 | 116 |
| Caries 5 | 60720 | 100 |
| Caries 6 | 57134 | 193 |
| Caries 7 | 57813 | 155 |
| Caries 8 | 61903 | 150 |
| Caries 9 | 57416 | 232 |
| Caries 10 | 58908 | 180 |
| Periodontitis 1 | 55873 | 265 |
| Periodontitis 2 | 58157 | 213 |
| Periodontitis 3 | 57991 | 227 |
| Periodontitis 4 | 58225 | 179 |
| Periodontitis 5 | 60048 | 120 |
| Periodontitis 6 | 58313 | 188 |
| Periodontitis 7 | 60354 | 217 |
| Periodontitis 8 | 58144 | 270 |
| Periodontitis 9 | 57435 | 140 |
| Periodontitis 10 | 60161 | 190 |
| Comorbid diseases 1 | 60018 | 244 |
| Comorbid diseases 2 | 59287 | 289 |
| Comorbid diseases 3 | 56799 | 254 |
| Comorbid diseases 4 | 58432 | 231 |
| Comorbid diseases 5 | 58400 | 263 |
| Comorbid diseases 6 | 61666 | 180 |
| Comorbid diseases 7 | 56683 | 254 |
| Comorbid diseases 8 | 58492 | 269 |
| Comorbid diseases 9 | 57497 | 274 |
| Comorbid diseases 10 | 57130 | 260 |

The clustering result statistics of OTU

As shown in the table, tag_num indicates the total number of tags and otu_num means that the otu is generated by clustering 97% sequence similarity in each sample.
